# Plant-microbe interactions in the phyllosphere: facing challenges of the anthropocene

**DOI:** 10.1038/s41396-021-01109-3

**Published:** 2021-09-14

**Authors:** Rosaëlle Perreault, Isabelle Laforest-Lapointe

**Affiliations:** 1 Département de biologie, Université de Sherbrooke, Sherbrooke, QC J1K 2R1 Canada; 2 Centre Sève, Université de Sherbrooke, Sherbrooke, QC J1K 2R1 Canada

**Keywords:** Climate-change ecology, Microbial ecology, Community ecology, Microbial ecology, Microbiome

## Abstract

Global change is a defining feature of the Anthropocene, the current human-dominated epoch, and poses imminent threats to ecosystem dynamics and services such as plant productivity, biodiversity, and environmental regulation. In this era, terrestrial ecosystems are experiencing perturbations linked to direct habitat modifications as well as indirect effects of global change on species distribution and extreme abiotic conditions. Microorganisms represent an important reservoir of biodiversity that can influence macro-organisms as they face habitat loss, rising atmospheric CO_2_ concentration, pollution, global warming, and increased frequency of drought. Plant-microbe interactions in the phyllosphere have been shown to support plant growth and increase host resistance to biotic and abiotic stresses. Here, we review how plant-microbe interactions in the phyllosphere can influence host survival and fitness in the context of global change. We highlight evidence that plant-microbe interactions (1) improve urban pollution remediation through the degradation of pollutants such as ultrafine particulate matter, black carbon, and atmospheric hydrocarbons, (2) have contrasting impacts on plant species range shifts through the loss of symbionts or pathogens, and (3) drive plant host adaptation to drought and warming. Finally, we discuss how key community ecology processes could drive plant-microbe interactions facing challenges of the Anthropocene.

## Introduction

The Earth is undergoing radical changes such as habitat loss, rising atmospheric CO_2_ concentration, increased frequency of extreme weather events, global warming, and higher risk of drought. Moreover, the intensification of anthropogenic activities has accelerated the impact of urbanization, land-use change, and pollution, modifying dramatically both terrestrial and marine ecosystems [1, 2]. For example, land-use change has been forecasted to cause major losses of habitat leading to the imperilment of thousands of species [3]. This loss of biodiversity will alter the delivery of ecosystem services that are crucial for human population health worldwide [4].

**Fig. 1:**
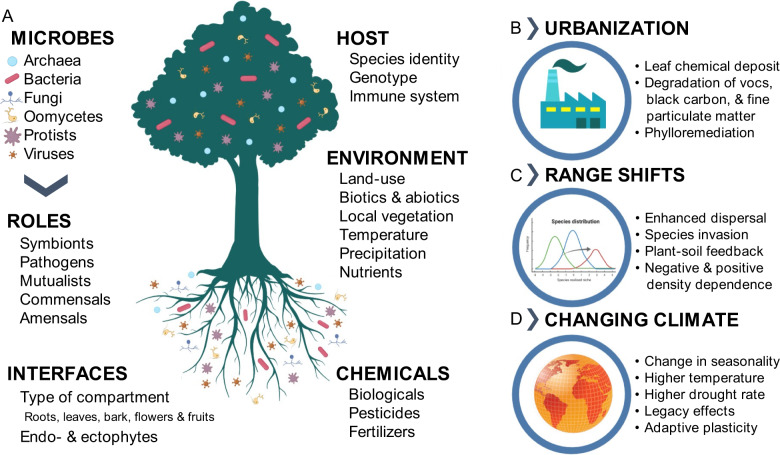
Plant-microbe interactions are involved in and impacted by global change. **A** A summary of characteristics and drivers of plant-microbe interactions. Plant-microbe interactions are impacted by abiotic and biotic stresses related to global change: (**B**) urbanization, (**C**) range shifts, and (**D**) climate change.

Microorganisms represent a massive diversity, colonizing soil, plants, and animals [5–7]. Although microbes have mainly been studied for their role as pathogens, advances in high-throughput sequencing techniques have rapidly improved our understanding of the beneficial roles of microbes for hosts and ecosystems [5–7]. Plant-microbe interactions (Fig. 1A) involve a great variety of microbes from multiple kingdoms [8, 9]. Plant microorganisms are further defined by host species [10], compartment, and tissue location [11]. Among the beneficial impacts of plant-microbe interactions, many studies have demonstrated the role of root microbiota in promoting plant growth and resistance to biotic and abiotic stresses [12, 13]. Leaf-associated microorganisms have also been shown to influence host fitness and growth [14, 15], resilience to abiotic stresses [9], and resistance to pathogens [16]. Furthermore, positive correlations have been found between the diversity of tree-associated microbiota and ecosystem productivity [17], and decreases in diversity have been correlated with disease state and disease propagation [18]. These findings stress the importance of understanding the mechanisms that could allow host-microbe interactions to drive the adaptation of terrestrial ecosystems to global change.

A striking trademark of the Anthropocene is the fact that most biomes have now been perturbed due to direct habitat modifications and indirect effects of global change on abiotic conditions. Biodiversity and function losses are threatening ecosystems and their inhabitants, including humans [19–21]. In this review, we summarize evidence of the role of plant-microbe interactions for host survival and fitness in the context of global change, with a particular focus on phyllosphere microbiota. Our discussion is structured around three key topics: urbanization, range shifts, and changing climate (i.e., rising temperatures and drought). Although we do not discuss here the impacts of synthetic chemicals (e.g., pesticides, pharmaceuticals) and biologicals (i.e., medicines grown and purified from large-scale cell cultures of bacteria or yeast, or plant or animal cells), these products influence plant-microbe interactions thus warranting more research effort on this important topic [22]. In short, we highlight promising research findings hinting at roles of plant-microbe interactions in (1) contributing to the remediation of urban pollution, (2) impacting plant species range shifts, and (3) improving plant host adaptation to harsher abiotic conditions. Finally, we briefly explore the interplay between plant-microbe interactions and global change in terms of community ecology processes.

### Urbanization

The expansion of urban centers and the anthropogenic activities within them are an immense source of various airborne pollutants [23]. These chemicals, as well as additional macro and micronutrients, are enriched on urban tree leaves compared to non-urban trees [24, 25], thus potentially impacting the dynamics and functions of plant-microbe interactions (Fig. 1B). Land-use type (e.g., cities, rural areas) as well as local vegetation, influence the composition of airborne microbial communities [26, 27]. Most importantly, anthropogenic activities have substantial impacts on plant microbiota, which can in turn remediate air pollutants [28] (i.e., degrading leaf-deposited chemicals) and influence human population health [29]. In this section, we review evidence that leaf microbial communities could play an important role in urban phylloremediation through degradation of pollutants such as ultrafine particulate matter, black carbon, and atmospheric hydrocarbons.

Phyllosphere bacterial and fungal community composition have been found to diverge significantly between urban and non-urban trees [24, 30, 31]. In two distinct studies comparing three sites across a gradient of urbanization in Europe and North America respectively, Imperato et al. [25] and Laforest-Lapointe et al. [31] observed a shift in community composition and a 10% higher bacterial alpha-diversity on tree leaves in urban areas. In another study, Espenshade et al. [32] did not observe an increase in alpha-diversity in cities, but they did detect an impact of urbanization (i.e., urban density and traffic patterns) on tree leaf bacterial community composition. Most interestingly, this shift was correlated with striking differences in ultrafine particulate matter and black carbon on tree leaves [32]. For fungal communities, Jumpponen and Jones [24] observed a lower diversity and richness on urban tree leaves, while Imperato et al. [25] observed a higher fungal load on city trees. Finally, Smets et al. [30] found that traffic levels have a significant impact on phyllosphere microbiota community composition. Together, these findings underline the need (1) to better define the elements that modulate variation of urban phyllosphere microbial communities (see Wuyts et al. [33]) and (2) to test if the detected changes in microbial taxonomic composition are also reflected in microbial functions.

Recent research has started to link genetic and functional research to microbial ecology, bringing evidence of the impact of urbanization on gene selection in the phyllosphere microbiota. For example, Imperato et al. [25] found a higher number of bacteria possessing genes encoding enzymes with predicted aromatic degradative activity and properties beneficial to plants (i.e., plant growth promotion) on leaves from an untouched forest than from urban areas. In addition, both air pollution and plant host species identity influence the amount of human pathogenic genes in phyllosphere microbiota [34]. This result suggests that specific plant species could be used in green spaces to reduce the number of pathogenic genes in urban environments [34]. It has also been shown that the prevalence of atmospheric hydrocarbons in cities (derived mostly from fossil fuel combustion) could favor the selection of hydrocarbon degrading bacteria by leaf microbes [35]. These contrasting findings support the need for future research investigating the influence of urban environments on microbial air pollution degradation capacity.

*Phytoremediation* is the use of plants to remediate a site contaminated with pollutants. Plant-microbe interactions have been suggested to be key for effective phytoremediation. Indeed, endophytes can improve phytoremediation in contaminated soils and water as well as the fitness and adaptation of associated plants in those conditions [36, 37]. Also, the presence of contaminants can result in higher prevalence of endophytes possessing catabolic genes in the bacterial community in a contaminant dependant manner [38]. This phenomenon can be artificially augmented, as Barac et al. [39] showed that the introduction of a plasmid encoding a toluene-degrading enzyme to a plant endophytic bacterium enhanced toluene degradation, thus reducing phytotoxicity and toluene evapotranspiration through the leaves by 50–70%.

The term *phylloremediation* was introduced by Sandhu et al. [40] when they reported direct evidence for volatile organic compound degradation by endophytic bacteria in the phyllosphere. Since then, multiple studies have provided evidence of pollutant uptake by leaf surfaces, as well as describing how bacteria augment this process by promoting plant growth or by degrading pollutants through specific metabolic pathways [41]. De Kempeneer et al. [28] demonstrated that toluene remediation is performed by phyllosphere microbiota through toluene-degrading bacteria. Although there is accumulating evidence of the potential of leaf microbes to impact urban pollution, much remains to be done to identify (1) what is the relative importance of phylloremediation compared to other mechanisms of pollution degradation; (2) which plant species are the most efficient at pollutant degradation and at reducing pathogenic genes; or (3) which microbial strains/plasmids can optimize air pollution degradation by the urban phyllosphere.

### Range shifts

Global change causes climatic conditions to shift. There are two main triggers of shifting ranges: (1) introduction of species to new habitats by human activities [42] and (2) environmental changes such as warming leading species to expand their range and colonize new environments where they could not survive before [43] or to contract their range because of increased biotic and abiotic stresses. From the perspective of plant-microbe interactions, encompassing both the macroscopic host and its interacting microbiota, invasive plant species could have unforeseen impacts on ecosystems through their associated microorganisms. In this section, we review how plant-microbe interactions can influence plant range shifts (Fig. 1C) and thus terrestrial ecosystem composition and function in the context of global change.

Elevation gradients provide practical systems in which to study the relative influence of biotic and abiotic factors on species distributions, community composition, and host-microbe specialization (where specialized interactions are optimal for host fitness [44]). For tree species along an elevation gradient, Cobian et al. [45] found that leaf fungal endophyte specialization followed a parabolic relationship, where specialization was at its highest at the center of tree species’ range (compared to the edges) and decreased closer to lower and upper range limits. Balint et al. [46] showed that the leaf fungal community of balsam poplars had higher diversity and evenness when trees were relocated to the upper edge of the elevation gradient. In this study, this higher evenness was hypothesized to be directly related to higher abiotic stresses at the northern range edge [46]. In comparison, Vacher et al. [47] showed that leaf bacterial communities vary less than fungal communities along elevation gradients, potentially because fungi are more sensitive to temperature. Plant community dynamics are also known to shift from competition to facilitation along elevation gradients, with negative interactions between plants decreasing with higher abiotic stresses [48]. Together, these results suggest that specialization would be reduced at a species range edge, but more work is needed to determine if this shift in interactions has a positive or negative impact on plant hosts.

Although, much of our review focuses on leaf microbes, we could not address species range shifts without mentioning the role of soil microbes on plant fitness. Plants influence soil biotic and abiotic parameters, which feed back to impact their growth and survival. This phenomenon is referred to as *plant-soil feedback* (PSF) [49]. PSF, and particularly its biotic components (i.e., soil microorganisms), are known to mainly have a negative effect on native species [50]. PSF drives species relative abundance (through conspecific negative density dependence) and therefore contributes to the regulation of plant diversity [51]. Species range expansion introduces plant species to novel soil biota, thus reinitializing previously established plant-microbe interactions. Soil biota can influence range shifts through multiple mechanisms including the loss of symbionts or novel positive interactions [52]. For example, Callaway et al. [53] found that *Centaurea maculosa* maintains negative interactions with soil biota in its native range but cultivates positive microbial feedbacks in North America, where it has been introduced. Contrastingly, Brown and Vellend [54] showed that the germination and survival of *Acer saccharum* seeds grown in soil from beyond its range limit was reduced, even if abiotic conditions were adequate for growth. The authors also observed a higher presence of fungal pathogens on seedlings grown in soil beyond range limit [54]. In addition, Carteron et al. [55] showed that soil biotic interactions have a strong influence on *A. saccharum* seedling performance, with the loss of fungal symbionts beyond the range likely slowing the species’ range expansion.

The *enemy release hypothesis* states that the ability of non-native species to colonize novel environments is boosted by the absence of natural enemies from its native distribution [56]. Indeed, multiple studies have found that invasive plants are significantly less colonized by common soil, floral, and foliar pathogens [57, 58]. Diez et al. [59] also reported evidence for PSF becoming more negative over time, although the authors could not differentiate pathogen influence from other variables. Plant-microbe interactions can impact species range shifts through a variety of microbial interactions. Yet, more studies providing a complete portrait of plant-microbe interactions (i.e., including both soil and leaf microbes as well as multiple kingdoms) are needed to tease apart how the contrasted roles of microbes balance out in driving plant range shifts.

### Changing climate

Most organisms across the Earth are currently experiencing rising temperatures [60]. Work by O’Brien and Lindow [61] showed that temperature can influence specific molecular pathways in bacteria colonizing plant leaf surfaces. Indeed, microbes can sense and respond to drastic changes in ambient temperatures [62], but the influence of long-lasting ambient warming is still largely unknown. Yet, recent articles have reviewed the impact of rising temperatures on specific interactions of pathogenic [63] and beneficial microbes [64] with their hosts, as well as on plant immune systems [65] and for soil microbes [66]. The following section will focus on the impact of a changing climate on plant-microbe interactions (Fig. 1D) by disrupting seasonal dynamics and increasing water limitation.

Several studies have demonstrated that temperature is one of the main drivers of soil microbial [67], phyllosphere fungal [68] as well as ectomycorrhizal fungi [69] community composition. The effects of intra-annual temperature variation have also been studied via seasonality, which is thought to be a key determinant of microbial community composition in soil, the rhizosphere [70], and the phyllosphere [24, 71–73]. Different abiotic factors vary with seasonality and therefore have a role in shaping microbial communities. For example, the number of days of frost in spring was found to be one of the main factors explaining phyllosphere fungal assemblage dissimilarities [68]. Peñuelas et al. [71] reported that the richness and evenness of the bacterial and fungal phyllosphere communities were lower under the harsh environmental conditions of the Mediterranean summer, compared with spring and winter. Additional studies have shown that the phyllosphere and root microbial communities are extremely variable over the growing season, but that clear predictable patterns in community composition could still be detected [74, 75]. Furthermore, Grady et al. [73] identified core leaf bacterial and archaeal communities for early, mid, and late phases of switch grass growing season. These observations suggest an influence of seasonality in driving leaf microbial community assembly, and the likelihood of functional and evolutionary changes associated with these predictable patterns. As seasonal trends are expected to shift with global change [76], this could have significant impacts on plant microbial community temporal composition.

Drought events threaten world food security through their devastating effects on essential crops [77]. It is thus important to identify plant-microbe interactions supporting crop productivity from future drought events [78]. If some studies have looked at the role of leaf microbes for plant resilience to drought, most of the literature has focused on soil microbes. Bacterial and archaeal soil community composition has been shown to vary considerably between arid, semi-arid, and Mediterranean climates, suggesting that water availability shapes communities across different ecosystems [79]. Drier climatic conditions have been associated with an increase in soil fungal diversity (i.e., evenness) and total abundance [80, 81]. In contrast, soils with a history of water stress display lower bacterial diversity [82]. Indeed, even though warming increases bacterial abundance under normal precipitation patterns, drought combined with warming causes significant decreases in bacterial abundance, when water availability becomes limiting [83]. Prolonged warming has also been shown to lead to *apparent thermal acclimation* of soil and ecosystem respiration, as microbial communities shift from cold-adapted to warm-adapted profiles [84]. However, the combined effects of warming and drought on microbial growth remains to be determined. Legacy effects of drought-adapted microbiota have been shown for plants subjected to subsequent water stress [80, 82].

For phyllosphere microbiota, it is well known that some endophytes improve host drought resilience (e.g., *Lolium* sp. and endophyte *Epichloë* [85]). Yet, few whole-community studies have looked at the impact of drought on phyllosphere microbiota. Nonetheless, higher richness of leaf fungal and bacterial communities, as well as higher diversity of nitrogen fixing bacteria, have been observed on trees submitted to experimental field droughts [71, 72]. It was also observed that drought differentially affected the functionality of root and leaf microbiota [86]. As global change accelerates, these findings highlight the need to improve our understanding of how plant-microbe interactions maintain terrestrial ecosystem productivity in the face of prolonged warming and drought.

### Through the lens of community ecology processes

**Fig. 2:**
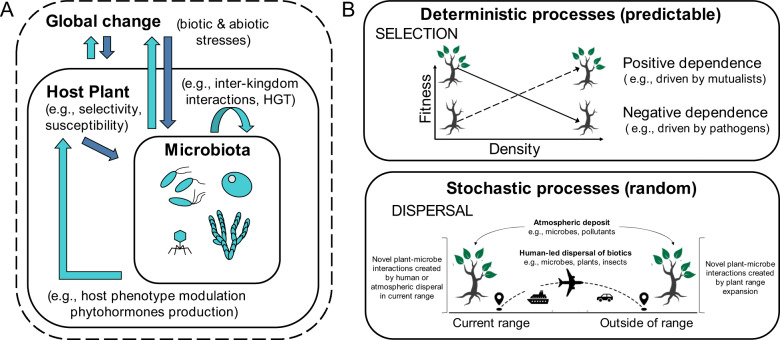
Tripartite interactions and microbial ecology. **A** Complex tripartite interactions drive feedbacks between global change, the host plant, and its leaf and root microbiota. **B** Examples of key community ecology processes providing unique insights on the interplay between plant-microbe interactions and global change.

A great challenge for microbial ecologists is to adapt community ecology theory to understand how microbial community processes drive patterns of assembly and function [87–91]. Several studies of plant-microbe interactions in the phyllosphere have attempted to quantify the relative roles of deterministic and stochastic processes in driving microbial community assembly and diversity with the aim of understanding their impact on ecosystem functions [10, 17, 31, 47, 68, 74, 92–101]. The *Synthesis of Community Ecology* champions four processes: selection, dispersal, drift, and speciation [102]. In this final section, we delve into community ecology to improve our understanding of tripartite interactions between global change, host plants, and microbial communities (Fig. 2).

Selection (i.e., distinct fitness among species leading to predictable demographic patterns) is a dominant deterministic process driving microbial community assembly. In the context of increasing biotic (e.g., arrival of a new competitor) and abiotic stresses (e.g., increase in drought frequency), selection could favor plants with a positive balance of host-microbe interactions (i.e., a predominance of interactions with beneficial microbes rather than with pathogens). This shift towards facilitation could modify plant species fundamental and realized niches. As plant species potentially expand their range, the loss of pathogens or symbionts could have species-specific impacts on conspecific density dependence. Unraveling the effects of global change on this complex dynamic is key to predict how plant species will adapt to a changing world.

Stochastic processes have also been argued to play a dominant role in driving microbial species abundance [91]. Dispersal (i.e., the movement of organisms across space) is increased by anthropogenic activities and thus gives plants and especially microbes chances to colonize new habitats. For microbial ecologists, this process is key as microbes can persist in dormant forms that allow for extensive spread over space and time. Dispersal can also lead to priority effects (i.e., species arrival order and timing during community assembly), a process that affects species abundances at multiple spatial scales. Although dormant microbes do not contribute directly to ecosystem processes, they could become important for community resilience and for the creation of novel plant-microbe interactions (potential diversity). In comparison, drift (i.e., demographic stochastic changes), has been suggested to play a weak role for microbial communities since declines in community size do not have a similar impact on microbial reproductive success. Speciation (i.e., the creation of novel species) is a complex process to study in microbial communities. The combination of extended dispersal, fast growth rates, and bacterial horizontal gene transfer could be important assets facilitating microbial adaptation. Improving our knowledge of the deterministic and stochastic processes driving root and leaf microbiota is crucial to predict the impacts of global change on terrestrial ecosystems.

### Future directions

In this review, we highlight properties of plant-microbe interactions facing challenges of the Anthropocene. Plant-microbe interactions have been shown to (1) contribute to remediating urban pollution, (2) impact plant species range shifts, and finally (3) improve plant host adaptation to drought and rising temperatures. These findings suggest that plant microbiota could have an underappreciated impact on terrestrial ecosystem biodiversity and productivity as global change continues in the decades ahead. Harnessing the potential of plant microbiota to support ecosystem services requires studying the role of inter-kingdom interactions (e.g., bacteria-phages, bacteria-fungi) through the lens of community ecology. Future research should investigate the rising impacts of synthetic chemicals and biologicals since these products are agents of global change [103]. In this review, we provide evidence that the field of microbial ecology is primed to offer ground-breaking resolutions of the roles of plant-microbe interactions in driving terrestrial ecosystems adaptation in the Anthropocene.
